# Immunological Impact of Oncolytic Adenoviruses On Cancer Therapy: Clinical Insights

**DOI:** 10.1002/eji.70024

**Published:** 2025-07-28

**Authors:** Reza Nadafi, Wenliang Dong, Victor W. van Beusechem

**Affiliations:** ^1^ ORCA Therapeutics B.V. 's‐Hertogenbosch The Netherlands; ^2^ Amsterdam UMC Location Vrije Universiteit Amsterdam Medical Oncology Amsterdam The Netherlands; ^3^ Cancer Center Amsterdam Cancer Biology and Immunology Amsterdam The Netherlands; ^4^ Amsterdam Institute for Immunology and Infectious Diseases Amsterdam The Netherlands

**Keywords:** immune response, immunogenic cell death, Immunotherapy, tumor microenvironment, tumor‐specific response

## Abstract

Oncolytic immunotherapy, particularly using engineered adenoviruses, has emerged as a promising approach in cancer treatment due to its dual mechanism of action: selective tumor‐cell destruction and inducing potent antitumor immune responses. This review focuses on the immunological effects observed in clinical trials involving conditionally replicating oncolytic adenoviruses (OAds), either with or without transgenes. These viruses primarily exert antitumor effects through mechanisms like direct oncolysis, apoptosis, necroptosis, and autophagy, while also activating innate and adaptive immune responses. Different genetic modification strategies have been employed to enhance the safety and therapeutic efficacy of OAds. However, these alterations may influence viral replication dynamics, oncolytic potency, and the duration of viral presence (i.e., persistence) within the tumor. Clinical data have shown that OAds can also profoundly alter the tumor microenvironment (TME), converting cold tumors to hot by increasing immune cell infiltration and activation. This conversion not only correlates with improved clinical outcomes but also creates conditions conducive to the efficacy of other immunotherapies, particularly immune checkpoint inhibitors (ICIs), which traditionally show limited activity in cold tumors. The synergistic potential of combining OAds with ICIs has shown promising results in improving clinical response rates. However, maximizing therapeutic benefit requires careful consideration of the OAd's immune‐activating capabilities and optimal timing of combination strategies. This review provides critical insights into the current state of OAd‐based immunotherapy, examining its role in modulating the TME, while addressing the complex interplay between oncolytic activity and sustained immune stimulation in clinical practice.

## Graphical abstract

Conditionally replicating oncolytic adenoviruses (OAds) mediate cancer cell destruction while triggering robust immune responses. Clinical data reveal their ability to remodel the tumor microenvironment and enhance responsiveness when combined with other therapies. This review highlights immune‐modulating effects of OAds in patients, with a focus on therapeutic implications and combination strategies.

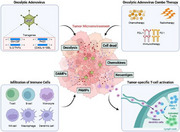

## 1 Introduction

Oncolytic immunotherapy, a promising therapeutic strategy in cancer treatment, utilizes engineered viruses that selectively infect and lyse tumor cells. This approach has captivated scientists for over a century, stemming from early observations of virus‐induced tumor regressions [1]. Among the various successfully applied oncolytic viruses (OVs), adenoviruses (Ads) have shown significant promise due to their safety, unique biological properties, and adaptability for genetic modification.

The antitumor activity of engineered oncolytic adenoviruses (OAds) is initially driven by direct tumor cell lysis [2]. This has been extensively demonstrated in preclinical models, including both in vitro cultures and xenograft mouse models, where OAds efficiently replicate and destroy tumor cells, even in the absence of immune cells [3]. These studies highlight OAds’ intrinsic cytolytic capacity, especially relevant in immunologically cold tumors that are typically unresponsive to immune checkpoint therapies [4]. However, while valuable, these preclinical models often fail to capture the full complexity of human tumors, especially in terms of heterogeneity, and antiviral responses, raising challenges for direct clinical translation.

Beyond direct lysis, OAds can induce immunogenic cell death (ICD), leading to the release of tumor‐associated antigens (TAAs), pathogen‐associated molecular patterns (PAMPs), and damage‐associated molecular patterns (DAMPs) [5]. These signals can recruit and activate antigen‐presenting cells, promote T cell priming, and potentially convert cold tumors into immunologically active ones [6]. This broader immune activation induced by OAds may lead to controlling distant tumor sites and contribute to sustained immune surveillance [3]. The combination of direct oncolysis and immune system engagement positions OAds as a promising strategy for tumors resistant to conventional immunotherapies.

Achieving clinical efficacy of treatment with OAds is influenced by several factors, including the route of administration, which plays a crucial role in viral distribution, replication, and immune activation [7, 8]. Additionally, combination strategies that integrate OAds with conventional therapies have demonstrated synergistic effects by disrupting the immunosuppressive tumor microenvironment (TME) and weakening the defenses of cancer cells [9, 10]. Moreover, advanced genetic modifications, including the incorporation of immunostimulatory transgenes, offer new opportunities to further potentiate OAds’ therapeutic effects [11]. Together, these factors shape the therapeutic outcomes of OAds in clinical trials, offering a path toward more effective and personalized cancer treatments.

In this review, we focus exclusively on human clinical trials involving engineered OAds to provide an overview of the immunological changes observed in patients across various cancer types. We examine how OAds, either with or without transgenes or used in combination with other therapies, affect immune cell populations in tumors and peripheral blood, and assess whether these modifications lead to improved clinical outcomes. By reviewing clinical data, we aim to clarify the current understanding of OAd‐induced immune modulation and guide future directions in oncolytic immunotherapy.

## 2 Adenovirus‐Induced Immunogenic Cell Death

**FIGURE 1:**
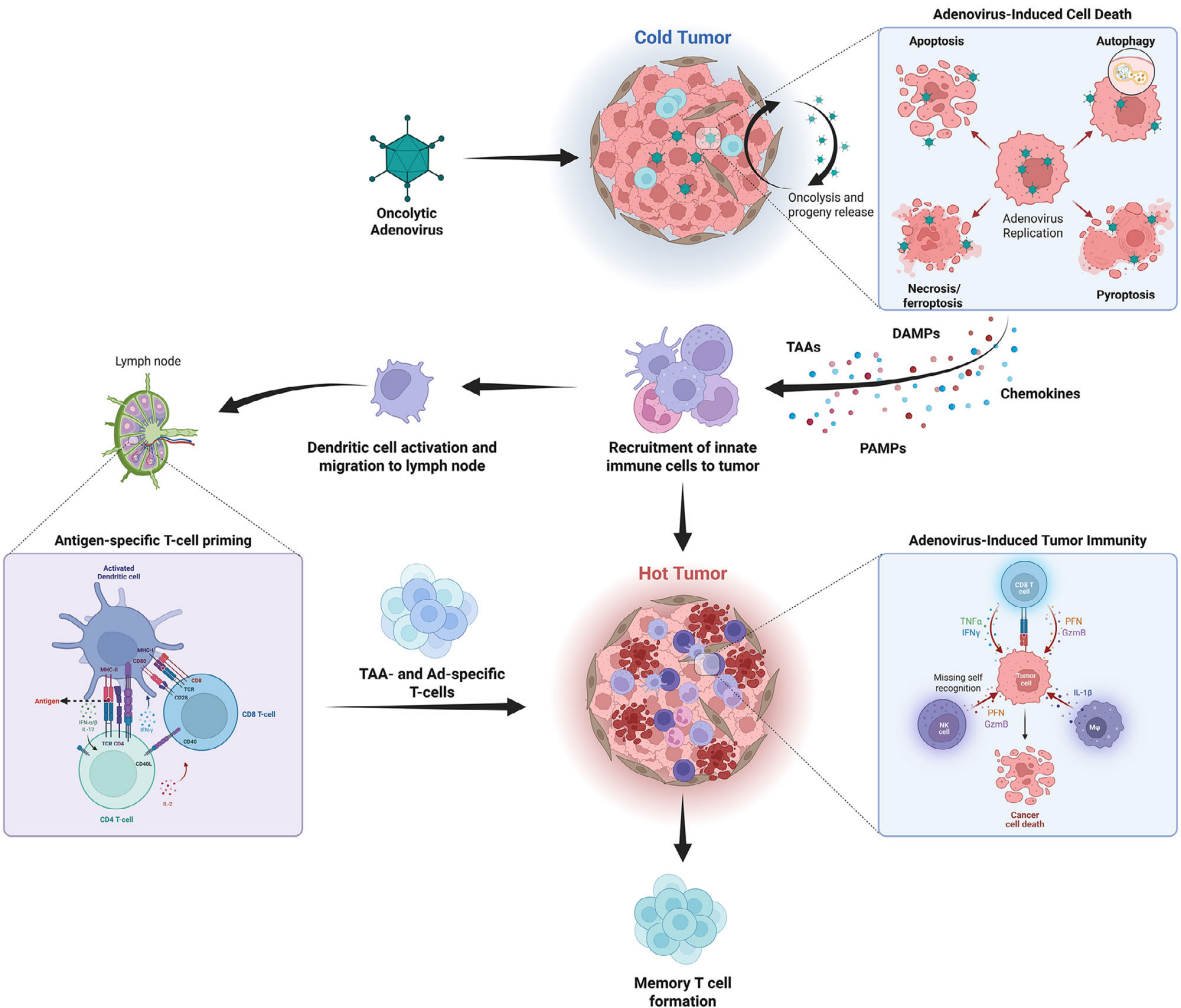
Oncolytic adenovirus‐induced tumor immunity. Upon infecting tumor cells, the virus replicates and induces oncolysis through various cell death mechanisms, including autophagy (self‐digestion), apoptosis (programmed cell death), necroptosis (inflammatory cell death), pyroptosis (inflammatory lysis), and ferroptosis (iron‐dependent cell death). This results in the release of immunogenic components such as damage‐associated molecular patterns (DAMPs), pathogen‐associated molecular patterns (PAMPs), chemokines (e.g., CXCL10), and tumor‐associated antigens (TAAs), including neoantigens. These signals recruit innate immune cells to the tumor and activate dendritic cells, which migrate to lymph nodes to prime TAA‐ and virus‐specific T cells. These T cells then infiltrate the tumor and mediate tumor‐cell destruction, converting the tumor from a "cold" to a "hot" tumor. This process may ultimately lead to the formation of memory T cells, providing long‐term immune protection against tumor recurrence.

Non‐malignant cells eliminate viruses by activating signaling pathways, while abnormalities in malignant cells allow survival and replication of viruses, resulting in oncolysis. OAds induce cell death [12] via mechanisms like autophagy [13, 14], apoptosis [15, 16], necroptosis [17, 18, 19], pyroptosis [20, 21], and ferroptosis [22] (Figure 1). This tumor cell lysis releases highly immunogenic components such as TAAs, PAMPs, and DAMPs, triggering immunogenic cell death (ICD) [23]. ICD activates both innate and adaptive immune cells, potentially enhancing immune cell infiltration into the TME and promoting the recognition of tumor cells by cytotoxic T lymphocytes [24, 25]. Additionally, the release of viral progeny and TAAs, including neoantigens, primes dendritic cells (DCs) to present these antigens to T‐cells, fostering tumor‐specific adaptive immune responses. This process can lead to tumor immunization, enabling the immune system to recognize and eliminate tumor cells. Through this dual mechanism of direct tumor lysis and immune activation, OAd‐based immunotherapy can target not only primary tumor sites but also distant metastases, establishing immunological memory and amplifying the anticancer effect, which is regarded as the most crucial aspect of oncolytic immunotherapy (Figure 1).

## 3 Adenovirus‐Induced Activation of Innate Immune Response

Cancer cell death that occurs through cell death pathways induced by OAds is accompanied by the release or membrane expression of highly immunostimulatory molecules from dying tumor cells. The extracellular release of high‐mobility group box 1 (HMGB1), calreticulin, extracellular adenosine triphosphate (ATP), and membrane expression of heat shock proteins (Hsp70 and Hsp90) act as alarmins and promptly activate the innate immune response in a nonspecific manner [26, 27, 28]. Additionally, Ad‐infected tumor cells secrete cytokines, including type I interferons (IFNs) and tumor necrosis factor α (TNF‐α), along with chemokines such as CXC‐chemokine ligand 10 (CXCL10), which all result in infiltration of innate immune cells such as DCs, macrophages, natural killer (NK) cells, and neutrophils to the tumor [5]. This cytokine and chemokine milieu creates a pro‐inflammatory environment that enhances the elimination of tumor cells. Upon recruitment, these innate immune cells become activated to contribute to the elimination of tumor cells.

Different DAMP molecules, such as ATP, HMGB1, and uric acid, or viral components released by Ad‐infected tumor cells, are recognized by different toll‐like receptors (TLRs) on DCs. This recognition activates and enhances antigen presentation by DCs. Activated DCs secrete inflammatory cytokines, such as IFN‐γ and IL‐12, which in turn activate adaptive immune cells, including CD8+ T‐cells, to target and eliminate tumor cells [5, 29, 30].

Macrophages play an important role in the elimination of tumor cells during oncolytic immunotherapy. When OAds infect tumor cells, they promptly attract inflammatory cells to the targeted lesions, substantially enhancing the infiltration of M1‐like macrophages within the tumor while reducing the presence of M2‐like macrophages. Clinical trials have demonstrated that OAds can shift tumor‐promoting M2 macrophages to tumor‐inhibiting M1 macrophages [31]. M1 macrophages then detect viral components through TLRs and other pattern recognition receptors [31, 32, 33]. This recognition triggers the production of pro‐inflammatory cytokines, including TNF‐α, IL‐6, and IL‐12, as well as the release of various chemokines like CXCL10, CXCL11, and CCL20 [34]. The cytokines and chemokines released by M1 macrophages create an inflammatory environment that attracts and activates other innate immune cells, such as DCs and NK cells [34]. Additionally, macrophages can phagocytose infected tumor cells and present tumor antigens to T‐cells, further promoting antitumor immunity and enhancing the overall cytotoxic response against tumor cells [35].

Virus‐infected tumor cells frequently downregulate human leukocyte antigen (HLA) class I molecules, reducing their recognition by T cells. However, NK cells can detect and target tumor cells independently of HLA expression [36]. This recognition triggers NK cell‐mediated cytotoxicity, leading to the direct killing of infected tumor cells through the release of perforin, granzymes, and the expression of TNF‐related factors such as FasL and TRAIL [36, 37]. Additionally, NK cells secrete inflammatory cytokines like IFN‐γ, which further enhance the activation and function of other immune cells, thereby amplifying the antitumor response [36]. Thus, the innate immune response plays a critical role in recognizing OAd‐infected tumor cells, initiating inflammation, and recruiting additional immune cells to effectively eliminate the tumor.

## 4 Adenovirus‐Induced Activation of Adaptive Immune Response

The adaptive immune response is critical for the long‐term elimination of tumor cells and prevention of recurrence. Viral antigens and TAAs released from OAd‐infected cancer cells are captured by activated DCs, which then migrate to secondary lymphoid organs such as lymph nodes (LNs), where they differentiate into mature DCs (Figure 1). These mature DCs express high levels of major histocompatibility molecules (MHC), co‐stimulatory molecules such as CD80 and CD86, and adhesion molecules, which are critical for priming and initiating effective antigen‐specific immune responses [5, 29, 30].

Activated antigen‐presenting DCs in the LNs present viral and tumor antigens to naïve T‐cells. The priming of antigen‐specific CD4+ and CD8+ T‐cell responses results in the expansion of T helper cells and cytotoxic T lymphocytes, respectively, which infiltrate the tumor sites and destroy tumor cells upon antigen recognition [5, 38]. CD8+ T cells play a crucial role in this process by recognizing antigens displayed on MHC class I molecules. Their cytotoxic action involves releasing perforin and granzymes, which induce apoptosis in target cells (Figure 1). Helper T‐cells also play a significant role by secreting cytokines that support the activation and proliferation of CD8+ T‐cells and other immune cells [39]. Consistent with this, depletion of T‐cells in a Syrian hamster in vivo tumor model has demonstrated that the therapeutic efficacy of Ads is largely mediated by T‐cells [40]. This coordinated response ensures a robust and sustained attack on the tumor cells, facilitating their complete eradication. Moreover, the interaction between the innate and adaptive immune systems is crucial for the formation of immunological memory [41]. Memory T‐cells, formed after the initial immune response, provide long‐term surveillance and rapid response to any residual or re‐emerging tumor cells [41, 42]. This ongoing immune vigilance significantly reduces the risk of cancer recurrence, enhancing the overall efficacy of OAd immunotherapy.

Beyond their direct antitumor effects, OAds also trigger antiviral immune responses. While antitumor immunity is obviously beneficial for cancer treatment, there is debate about whether antiviral immunity reduces or promotes the efficacy of OAd immunotherapy [43]. A robust antiviral response may limit viral replication and spread, thereby diminishing the direct oncolysis of cancer cells [44]. However, it should also be noted that OVs such as herpes simplex virus‐1 have shown antitumor therapeutic benefits in the presence of pre‐existing antiviral immunity [31, 44, 45, 46], supporting the clinical relevance of OVs even in patients with prior viral exposure.

Through the activation of both innate and adaptive immune responses, OAds provide a powerful and comprehensive strategy for eliminating tumor cells. By engaging diverse immune components, OAds not only directly kill tumor cells but also establish a long‐lasting immunological memory, reducing the risk of recurrence and improving therapeutic outcomes.

## 5 Immunological Impact of Oncolytic Adenovirus in Clinical Trials

The development of OAds, with or without transgenes, has progressed from preclinical models to clinical trials. These trials evaluate not only the efficacy of the engineered viruses but also their safety profiles and ability to induce beneficial immunological changes in the TME. In the following sections, we will review published clinical trials involving engineered OAds as highlighted in Tables 1 and 2. We will emphasize the immunological alterations triggered by these viruses in cancer treatment, considering both transgene‐enhanced and nonarmed variants. It is important to interpret these trial results in the context of the specific genetic modifications made to each OAd, recognizing the potential trade‐offs between enhanced tumor specificity, lytic replication rate, immune stimulation by transgene expression, and overall oncolytic potency.

**TABLE 1:** Immunological overview of a clinical trial involving oncolytic adenovirus without transgenes

Virus name	Indication (s)	NCT	Phase	Administration	Combination	Immunological summary	PMID
H101	Refractory malignant ascites	NCT04771676	Phase II	IP	—	IFNγ pathway genes upregulated Increased CD8+ T‐cell proliferation and cytotoxicity Elevated PD‐L1, PD‐L2, CD80 on macrophages PD‐1, CTLA4, and LAG3 on CD8+ T‐cells.	38659226
	Head and neck Esophagus	NCT03780049	Phase III	IT	Chemotherapy	No data on immune cell subsets in circulation or tumors	15601557
	Advanced cancers	—	Phase II	IT	Chemotherapy	No data on immune cell subsets in circulation or tumors	15534920
ICOVIR‐5	Solid tumors	NCT01844661	Phase I/II	IV	—	No significant changes in circulating immune cell subsets reported	32053771
	Melanoma	NCT01864759	Phase I	IV	—	No data on immune cell subsets in circulation or tumors	30234393
OBP‐301	Esophageal	NCT03213054	Phase I	IT	Radiotherapy	Increased CD8+ T‐cell infiltration, Elevated PD‐L1 expression in TME	34153720
	Advanced HCC	NCT02293850	Phase I	IT	—	Increased CD8+ T‐cell infiltration, Low PD‐L1 expression in TME	37060176
	Solid tumors	—	Phase I	IT	—	No changes in circulating immune cell subsets	19935775
DNX‐2401	Glioblastoma	NCT00805376	Phase I	IT	—	No changes in PD‐1+ or PDL‐1 expression. Slight increase in CD4+ and CD8+ T‐cell infiltration. Reduced TIM3+ cell infiltration	29432077
		NCT03178032	Phase I	IT	Radiotherapy Chemotherapy	Increased CD4+ and CD8+ T‐cell infiltration at relapse Reduced CD11b+ myeloid cells and CD4+/CD8+ T‐cells at autopsy. Increased CD163+ M2 macrophages at autopsy	35767439
		NCT02798406	Phase I/II	IT	Pembrolizumab	Increased CD3+, CD4+, CD8+ T‐cell, and CD68+ macrophage infiltration Increased TIGIT and LAG3 expression	37188783
		NCT01582516	Phase I	IT	—	Increased Th1 cytokines (IFNγ, TNFα). Increased CD56+ NK cells, DCs, CD4+, and CD8+ T‐cells in CSF. Intratumoral macrophages, CD4+, and CD8+ T‐cells elevated. No changes in circulating immune cell subset	35176144
Enadenotucirev	Solid tumors	NCT02053220	Phase I	IV	—	Increased CD8+ and CD8+PD‐1+ T‐cells in tumor nest. Increased CD4+ and Tregs in the stromal region. PDL‐1 expression inconclusive	28923104
	Solid tumors	NCT02028442	Phase I/II		—	No data on immune cell subsets in circulation or tumors	30691536
	Ovarian cancer	NCT02028117	Phase I		Chemotherapy	Increased CD8+Granzyme B+ T‐cell infiltration No change in CD4+ T‐cells, Ki67, or PD‐L1 expression	34893524
	Rectal cancer	NCT03916510	Phase I		Radiotherapy Chemotherapy	No data on immune cell subsets in circulation or tumors	32532291
	Solid tumors	NCT02636036	Phase I		Nivolumab	Enhanced CD8+ T‐cell infiltration in tumors and stroma Increased CD8+/FoxP3+ ratio, CD8+Granzyme B+ T‐cells, and activated CD8+ T‐cells (CD38, HLA‐DR) Th1 and related cytokines (IFNγ, IL‐12p70, IL‐17A)	37094988

Abbreviations: IP, intraperitoneal; IV, intravenous; IT, intratumoral; TME, tumor microenvironment; HCC, hepatocellular carcinoma; CSF, cerebrospinal fluid.

**TABLE 2:** Immunological overview of a clinical trial involving oncolytic adenovirus with transgenes

Virus name	Transgene	Indication (s)	NCT	Phase	Administration	Combination	Immunological summary	PMID
CG0070	GM‐CSF	Bladder cancer	—	Phase I	IVES	—	No data on immune cell subsets in circulation or tumors	23088985
			NCT02365818	Phase II	IVES	—	No data on immune cell subsets in circulation or tumors	28755959
ONCOS‐102	GM‐CSF	Solid tumors	NCT01598129	Phase I	IT	Chemotherapy	Enhancing CD8+ T‐cell infiltration Systemic tumor‐specific CD8+ T‐cell response. Upregulation of PD‐L1 on tumor cells. Induction of Th1‐type gene signature	26981247
		Melanoma	NCT03003676	Phase I	IT	Chemotherapy Pembrolizumab	Increased CD8+ and CD4+ T‐cell infiltration. Sustained upregulation of immune response‐related genes	36112545
		Pleural mesothelioma	NCT02879669	Phase I/II	IT	Chemotherapy	Increased CD4+, CD8+, and Granzyme B+ T‐cell infiltration. Increased CD8+/Treg ratio and M1/M2 macrophage polarization	37661097
TILT‐123	TNFα, IL2	Melanoma Solid tumors	NCT04695327	Phase I	IT/IV	—	Increase in CD8+ T cells and CD56+ NK cells. Increased CXCL9, CXCL10, CXCL11, IFNγ, TNFα in serum. Transient decrease in lymphocytes post‐therapy	38546220
		Ovarian cancer	NCT05271318	Phase Ia	IV/IT/IP	Pembrolizumab	Increase in CD8+PD‐1+ T‐cells and CD56+PD‐1+ NK cells. Increase in CD4+ T‐cells and B‐cells, Ad neutralizing antibodies correlated with clinical benefit	39910037
		Melanoma	NCT04217473		IT/IV	TILs	No changes in circulating immune cell subsets. Nonsignificant increased immune reactivity ex vivo	40107242
YSCH‐01	L‐IFN	Solid tumors	NCT05180851	Phase I	IT	—	No data on immune cell subsets in circulation or tumors	38719544
LOAd‐703	CD40L 4‐1BBL	Pancreatic cancer	NCT02705196	Phase I/II	IT	Chemotherapy	Increased Ad‐specific antibodies. Increase Ad‐specific T‐cells. Systemic increase in CD8+ effector memory T‐cells	38547893
VCN‐01	PH20	Retinoblastoma	NCT03284268	Phase I	IVT	—	Increased CD4+/CD8+ T‐cells. Localized inflammation, systemic antibody responses. Plasma cells in necrotic areas	30674657
		Pancreatic cancer	NCT02045589	Phase I	IV	Chemotherapy	No data on immune cell subsets in circulation or tumors	35149591
		Solid tumors Pancreatic cancer	NCT02045602	Phase I	IV	Chemotherapy	Th1 response induced in serum. Increased CD8+ T‐cell infiltration. Decreased Tregs. Upregulated PD‐1 and CTLA‐4 in tumor	35338084

Abbreviations: GM‐CSF, granulocyte‐macrophage colony‐stimulating factor; IVES, intravesical therapy; IV, intravenous; IT, intratumoral; IVT, intravitreal injection; TILs, tumor‐infiltrating lymphocytes; Ad, adenovirus.

### 5.1 Oncolytic Adenovirus without Transgene

Without transgenes, OAds form the basis of oncolytic immunotherapy, relying on the virus's natural lytic cycle to infect and destroy cancer cells. Various genetic modification strategies have been developed to create more cancer cell‐selective and safe OAds. ONYX‐015, the first engineered OAd in clinical trials (1996) [47], targeted p53‐deficient tumors via E1B‐55K deletion but also replicated in wild‐type p53 tumors [48, 49, 50, 51]. It showed a strong safety profile across intratumoral (IT), intravenous (IV), and intraperitoneal (IP) routes, with no maximum tolerated dose in hundreds of patients. Immune responses in the circulation or TME were minimally assessed in these trials [52, 53, 54, 55, 56]. As monotherapy, efficacy was limited, modest responses in head and neck cancer [47, 57], none in pancreatic, ovarian, or colorectal [58, 59, 60], highlighting the need for improved OAds and combination treatment strategies.

H101 (Oncorine) is very similar to ONYX‐015 and was tested in multiple clinical trials. A Phase II study found H101 combined with chemotherapy to be effective and safe for advanced cancer, while a Phase III trial demonstrated that this combination was more effective than chemotherapy alone for head and neck or esophageal cancers, showing potential enhanced antitumor efficacy [61, 62]. Oncorine was also tested in a Phase II trial for malignant ascites resistant to standard treatments [63]. The trial showed that IP administration of H101 is safe and effective, controlling ascites and stimulating strong activation of immune responses in samples obtained up to day 14 posttreatment [63]. Treatment activated IFN‐γ pathways, enhanced CD8+ T‐cell activity, and increased exhaustion molecules such as PD‐1, CTLA‐4, and LAG3 on cytotoxic CD8+ T‐cells [63].

Ad5‐Δ24 (an E1A‐CR2‐deleted OAd) and ICOVIR‐5 (a derivative of Ad5‐Δ24 with restricted E1A expression) are designed to replicate in cancer cells with defective retinoblastoma (RB) pathway [64, 65, 66, 67]. In a Phase I trial for metastatic melanoma, ICOVIR‐5 was well tolerated and reached melanoma metastases with a single IV infusion but did not cause significant tumor regression [68]. In a Phase I/II trial, autologous mesenchymal stem cells (MSCs) were used to deliver ICOVIR‐5 in patients with relapsed or refractory solid tumors [69]. The trial demonstrated that repeated IV delivery of ICOVIR‐5 via MSCs is safe and well‐tolerated in pediatric patients, effectively delivering OAd with minimal toxicity, though no significant changes in immune cell subsets were observed [69].

Telomelysin (OBP‐301) is an OAd with E1A driven by the human telomerase reverse transcriptase (hTERT) promoter [70]. Several clinical trials have been conducted involving OBP‐301. In a Phase I clinical trial combining OBP‐301 with radiotherapy for esophageal cancer, the treatment was well‐tolerated [71]. Immune activation, indicated by increased CD8+ T‐cell infiltration and elevated PD‐L1 expression, was observed in patients who exhibited a partial response 1 month after treatment [71]. In advanced hepatocellular carcinoma, OBP‐301 demonstrated safety and tolerability in a Phase I trial, with 78% of patients achieving stable disease. Despite modest overall efficacy compared with other therapies, the treatment improved local tumor control, with increased CD8+ T‐cell infiltration and low PD‐L1 expression [72]. However, none of the patients experienced obvious antitumor activity [72]. In another Phase I trial, OBP‐301 showed a favorable safety profile in various solid tumors, with one patient achieving a partial response [73]. No changes were observed in circulating lymphocytes [73].

Ad5‐Δ24.RGD (DNX‐2401) is an OAd derived from Ad5‐Δ24 carrying a cyclic RGD peptide motif inserted into its fiber capsid proteins [64]. This insertion boosts infectivity by targeting integrins on cancer cells. DNX‐2401 was tested in multiple clinical trials for the treatment of gliomas in which distinct yet consistent immunological changes were reported. In a Phase I study, DNX‐2401 triggered robust immune responses in recurrent high‐grade gliomas, with significant CD4+ T‐cell infiltration, a shift toward a Th1 response, and reduced expression of exhaustion markers like TIM‐3. The immune response persisted years after treatment, marked by tumor absence [74]. In another Phase I study, DNX‐2401 was combined with radiotherapy and chemotherapy for the treatment of pediatric diffuse intrinsic pontine glioma patients and showed that DNX‐2401 induced an initial surge in CD4+ and CD8+ T‐cells, along with proinflammatory macrophages [75]. At relapse, however, there was a shift toward immunosuppressive M2 macrophages and decreased T‐cell activity [75]. A Phase I/II study of combined DNX‐2401 with pembrolizumab, an anti‐PD‐1 antibody, in recurrent glioblastoma, revealed that in a few patients with a high objective response rate, a significant increase in immune infiltration of CD3+ and CD8 positive cells could be observed [76]. Postprogression analysis indicated adaptive immune resistance through upregulation of checkpoints like TIGIT and LAG3 [76]. In another Phase I trial, DNX‐2401 has been used in recurrent GBM, showing a marked increase in CD4+ and CD8+ T‐cells, NK cells, and Th1 cytokines (IFN‐γ, TNF‐α) in the cerebrospinal fluid [31]. High IFN‐γ levels correlated with better survival, suggesting its potential as a biomarker. Additionally, a shift from tumor‐supportive M2 macrophages to tumor‐suppressive M1 macrophages was observed [31], indicating sustained local immune activation. No changes were detected in the serum levels of cytokines and chemokines, nor the immune cell subsets present in the circulation [31]. Notably, this clinical trial resulted in long‐term survival in two patients, including one who remains alive more than eight years after treatment. Remarkably, while tumor regression in this patient was initially slow, the tumor disappeared on MRI scans after all supportive medication, including immune‐suppressive steroids, was withdrawn one year after virus infusion [31]. These outcomes underscore the potential of OAds to elicit a durable immunogenic antitumor response, even in heavily pretreated patients with advanced disease [31].

An improved variant of Ad5‐Δ24.RGD, ORCA‐010, has been engineered to enhance both its safety and therapeutic efficacy [67]. In addition to the Ad5‐Δ24 and RGD modifications, ORCA‐010 includes a potency‐enhancing T1 mutation [67]. The T1 mutation, specifically a single adenine insertion at position 445 within the E3/19K gene, disrupting its endoplasmic reticulum (ER) retention signal, facilitates increased release of viral progeny from infected cells. Preclinical studies with human cells have shown that ORCA‐010 not only exhibits enhanced oncolytic efficacy but also activates proinflammatory myeloid cells and increases the co‐stimulatory capacity of melanoma‐conditioned DCs [77, 78]. Unpublished data from our ORCA‐010 Phase I/IIa trial in prostate cancer (Clinical trial ID NCT04097002) suggest that it is safe and promotes local and systemic antitumor immune responses, supporting its potential as an immunotherapy.

Enadenotucirev (EnAd) is a chimeric virus that combines the Ad3 and Ad11p serotypes [79]. In preclinical studies, EnAd has shown effectiveness against different solid tumors [79]. It has been tested in multiple Phase I clinical trials where it was administered IV to patients for treating solid tumors, either as a monotherapy or in combination with other therapies [10, 80, 81, 82, 83]. During a Phase I trial involving various solid tumors, CD8+ T‐cells were notably present within the tumor nests of microsatellite‐stable colorectal cancer [80]. These CD8+ T‐cells, which also express PD‐1, may represent activated CTLs. In contrast, CD4+ T‐cells were predominantly located in stromal regions, along with the Treg subset, which may contribute to an immunosuppressive environment in nontumor stromal areas [80]. In another Phase I study, EnAd was combined with paclitaxel to treat platinum‐resistant ovarian cancer [10]. Biopsies taken 5 weeks after treatment showed a three‐fold increase in CD8+ T‐cell infiltration, with some of these cells being Granzyme B+, suggesting cytolytic activity. Although no clear correlation between T‐cell activity and overall survival was observed, a significant reduction in tumor burden was noted in the patient with the highest T‐cell increase to over 1000 CD8+ cells/mm^2^. No substantial effect on CD4+ T‐cells, or Ki67, PD‐L1, or PD‐1 expression was seen in posttreatment samples [10].

Additionally, EnAd has been combined with nivolumab, a PD‐1 inhibitor, in a Phase I study involving patients with advanced or metastatic epithelial cancers that were unresponsive to standard therapies [81]. This combination led to increased levels of Th1 cytokines and enhanced CD8+ T‐cell infiltration in both tumors and stromal regions in biopsies taken at 8–15 days posttreatment. The CD8+/FoxP3+ T‐cell ratio and CD8+ Granzyme+ T‐cells also increased, with stronger effects seen in higher‐dose cohorts [81]. Circulating immune cells showed signs of activation, including increased CD38 and HLA‐DR expression on CD8+ T cells. No clear relationship between immune activity and OS was identified [81].

### 5.2 Oncolytic Adenovirus Armed with Transgenes

To enhance the therapeutic and oncolytic potency of OAds, strategies focus on engineering them for cancer‐specific replication and transgene expression to boost oncolysis and immune responses. Most OAds involve partial or complete deletions of the E3 region to insert transgenes due to the genome size limitations of adenoviruses [84]. While transgene insertions can increase therapeutic potential, it has been demonstrated that deletion of the E3 region results in reduced oncolytic potency [85]. The E3 region of Ad5 encodes proteins critical for viral spread and immune modulation, such as E3‐gp19K (which inhibits MHC class I presentation), RID complex (which blocks apoptosis), and ADP (which promotes cell lysis). Removing the genes encoding these proteins may compromise viral replication and immune activation, potentially limiting oncolytic effects [85]. Therefore, careful design of E3‐deleted OAds is crucial to balance enhanced therapeutic benefits while retaining sufficient oncolytic potency. Numerous OAds with various modifications aimed at enhancing their oncolytic potency have been evaluated in preclinical investigations, with some progressing to clinical trials. Here, we will discuss some of the most advanced clinical trials that have used OAds armed with transgenes.

Granulocyte‐macrophage colony‐stimulating factor (GM‐CSF) is a cytokine that drives the production of myeloid cells like neutrophils, monocytes, macrophages, and DCs in response to pathological conditions [86]. By modulating innate immune cells that activate adaptive responses, GM‐CSF plays a crucial role in immune surveillance and supports antitumor immunity [86]. In clinical trials, IT injection of OAds engineered to express GM‐CSF may increase the number of DCs and T cells in the TME, while also enhancing their activation. In line with this, there are several OAds expressing GM‐CSF in clinical trials such as CG0070 and ONCOS‐102 [87]. In clinical trials for BCG‐unresponsive high‐risk nonmuscle invasive bladder cancer, CG0070 demonstrated promising response rates, with notable success in patients with carcinoma‐in‐situ (CIS) [88, 89]. The treatment was well tolerated, showing no severe adverse events, but lacked immunological data [88, 89]. These findings highlight the need for further research to evaluate long‐term efficacy and to better understand systemic immune cell activation.

In a Phase I dose‐escalation study, ONCOS‐12 was combined with cyclophosphamide and administered IT in patients with treatment‐refractory solid tumors [90]. ONCOS‐102 effectively stimulated antitumor immunity, resulting in increased CD8+ T‐cell infiltration and detection of a systemic tumor‐specific CD8+ T‐cell response to MAGE‐A3/A1 and N‐ESO‐1 antigens compared with baseline, which correlated with improved survival [90]. Additionally, ONCOS‐102 treatment led to the induction of a Th1 gene signature and upregulation of PD‐L1 expression on tumor cells [90]. In another Phase I study, the combination of ONCOS‐102 and pembrolizumab was well tolerated and demonstrated promising efficacy in advanced melanoma patients resistant to anti‐PD‐1 therapy [91]. Biopsies taken at up to week 9 posttreatment demonstrated an increased infiltration of CD8+ and CD4+ T‐cells and sustained upregulation of immune response‐related genes in patients who experienced disease control compared with those with disease progression [91]. It is noteworthy that tumors from patients with disease control exhibited higher baseline T‐cell infiltration, as indicated by CD3+, CD4+, and CD8+ T‐cell marker gene expression. Additionally, the continued presence of immune cell infiltration was associated with improved clinical outcomes [91]. In an explorative Phase I/II study, ONCOS‐102 was combined with pemetrexed and cisplatin/carboplatin for treatment of unresectable malignant pleural mesothelioma (MPM), a cancer with a notoriously poor prognosis [92]. ONCOS‐102 was well tolerated and did not increase chemotherapy‐related toxicities [92]. Importantly, ONCOS‐102 promoted a more immunogenic TME, leading to significant immune infiltration in tumor biopsies at 36 days posttreatment. This included increased infiltration of CD4+, CD8+, and granzyme+ T‐cells, and heightened expression of cytotoxicity‐related genes such as granulysin, granzymes, and perforin 1, which was not observed with chemotherapy alone [92]. Increased T‐cell infiltration and serum GM‐CSF levels were associated with better survival [92]. These trials highlight ONCOS‐102's potential to boost immune responses and survival in resistant cancers, enhancing outcomes with pembrolizumab in melanoma and chemotherapy in mesothelioma, but further research is needed to confirm their long‐term efficacy.

TILT‐123 is a human Ad5/3‐derived OAd designed for enhanced T‐cell activation. It features cancer‐specific replication controls and includes transgenes that encode IL‐2 and TNF‐α to boost T‐cell growth, trafficking, and tumor apoptosis [93]. In a Phase I dose‐escalation study, TILT‐123 has shown promising results as a monotherapy in solid tumors [94]. Treatment led to a transient drop (1‐2 days post‐treatment) in peripheral lymphocytes and increased CD8+ and CD56+ lymphocytes in tumors (days 8 and 36) [94]. Systemic effects included viral spread and lymphocyte infiltration in both injected and distant lesions, along with elevated levels of T‐cell trafficking chemokines like CXCL9, CXCL10, and CXCL11 and cytotoxic cytokines like IFN‐γ and TNF‐α [94]. While no tumor‐specific T‐cell responses were reported in this clinical trial, several patients exhibited antitumor activity on imaging and achieved prolonged survival [94]. TILT‐123 has also been evaluated in clinical trials in combination with immune checkpoint inhibitors and adoptive cell therapy, offering insights into its immunomodulatory capacity [95, 96]. In a Phase Ia trial in patients with platinum‐resistant ovarian cancer, IT administration of TILT‐123 with pembrolizumab increased infiltration of PD‐1+CD8+ T‐cells, PD‐1+CD56+ NK cells, CD4+ T‐cells, and B‐cells into the TME. Higher serum titers of anti‐Ad neutralizing antibodies (NAbs) were associated with clinical benefit [96]. In a separate trial involving metastatic melanoma patients’ refractory to checkpoint blockade, TILT‐123 combined with tumor‐infiltrating lymphocytes (TIL) therapy was well tolerated, with most immune populations remaining stable during treatment [95]. Notably, immune cells isolated on day 8 exhibited significantly elevated PD‐1 expression [95]. Ex vivo analysis indicated a non‐significant trend toward increased immune reactivity to autologous tumors in some patients [95]. Collectively, these findings highlight the potential of TILT‐123 to modulate the TME, enhance immune activation, and synergize with other immunotherapies.

Secondary signals involving costimulatory molecules on immune cells are critical for activating cellular and humoral responses. Among these, CD40L/CD40 interactions impact DCs, B‐cells, and other cell types [97], and 4‐1BB enhances immunological memory and NK cell expansion [98]. The OAd LOAd703, engineered to express CD40L and 4‐1BBL, has been combined with chemotherapy and tested in a Phase I/II trial for advanced pancreatic cancer [99]. LOAd703 rapidly increased Ad‐specific antibodies, and boosted Ad‐specific T‐cells, and circulating CD8+ effector memory T‐cells; however, tumor‐specific CD8+ T‐cell responses were not evaluated in this clinical trial [99].

VCN‐01 is an engineered OAd derived from ICOVIR‐5 to replicate in cancer cells with defective RB pathway and to express PH20 hyaluronidase enzyme, which helps degrade the extracellular matrix, particularly the hyaluronic acid‐rich tumor stroma, facilitating better viral spread and enhancing the delivery of chemotherapy [100]. In a Phase I trial for refractory intraocular retinoblastoma, VCN‐01 caused localized inflammation in the eye and systemic antibody responses, with immune cell infiltration observed in necrotic tumor areas, while viable tumor regions had minimal immune cell presence [101]. Another Phase I study for pancreatic ductal adenocarcinoma (PDAC) showed VCN‐01's ability to disrupt the hyaluronan‐rich stroma, though detailed immunological data were lacking [102]. However, a different Phase I trial in which VCN‐01 was combined with chemotherapy in solid tumors revealed significant immune changes, including increased pro‐inflammatory mediators observed up to 8 days after treatment (IL‐6, IFN‐γ), a Th1 response with elevated IL‐18 and TIM‐3, and a rise in anti‐Ad NAbs [103]. Tumor biopsies at day 8 or 28 taken from 14/24 patients showed an increased CD8+ T‐cell infiltration, reduced Tregs, and upregulation of immune checkpoints like PD‐1 and CTLA‐4 in approximately 50% of the tumor biopsies [103]. These findings suggest VCN‐01 can disrupt the tumor stroma and boost the immune response. However, the duration and the tumor specificity of the immune responses are unclear.

## 6 Conclusion and Future Prospects

Oncolytic immunotherapy has established itself as a promising approach in cancer treatment due to its ability to induce direct oncolysis and robust immune responses. OAds, with or without transgenes, induce immunogenic cell death via apoptosis, necroptosis, or autophagy, releasing danger signals that recruit innate immune cells and activate adaptive immunity. This process can promote long‐term immunological memory, which is essential for preventing tumor recurrence [31].

To date, OAds have demonstrated favorable safety profiles and immune‐modulating effects in various clinical trials. Their immunogenic nature and ability to reshape the TME make them attractive candidates for combination therapies [104]. When paired with treatments such as immune checkpoint inhibitors (ICIs), adoptive cell therapies, cancer vaccines, or bispecific T‐cell engagers, OAds can initiate and amplify immune responses, particularly in cold tumors where ICIs alone are ineffective [4]. These combinations have shown synergistic effects, but optimal timing, sequencing, and vector design remain critical for success.

Despite these key advantages of OAds, several challenges persist. OAds often show limited persistence (typically 4–6 weeks) in patients [73, 103, 105], and engineering for improved specificity or immune activation can sometimes compromise their replication or spread [85]. Additionally, issues like pre‐existing NAbs, antiviral immunity, and off‐target effects continue to limit their systemic use in clinical trials [106]. Notably, recent findings indicate that incorporating an RGD motif into the viral capsid enables OAds to evade NAbs directed against the native Ad5 fiber [107]. In glioblastoma patients treated with Ad5‐Δ24.RGD, the RGD‐mediated alternative entry pathway conferred resistance to both pre‐existing and treatment‐induced anti‐Ad5 neutralization [31, 107]. This property highlights the potential of RGD‐modified OAds for repeated administration, particularly in the brain, where NAb‐mediated inhibition of RGD‐dependent infection appears minimal.

The route of administration also plays a key role in therapeutic outcomes. While IT delivery promotes local immune activation, it is restricted to accessible lesions [7, 8, 108]. IV injection allows systemic distribution but is hindered by rapid clearance and immune neutralization [7, 8]. Locoregional approaches (e.g., IP) or the use of cell carriers like mesenchymal or neural stem cells are being explored to improve delivery and enhance tumor targeting [7, 8, 109, 110].

Although clinical trials with immunological data have reported early conversion of cold TME into hot ones following treatment, limited data are available on the tumor‐specificity of this response or its long‐term sustainability. However, multiple studies indicate that durable clinical benefits, including sustained tumor shrinkage, are frequently observed in patients with high levels of immune infiltration and activation [76, 111].

Advancing our understanding of the immunobiology underlying oncolytic immunotherapy is crucial for unlocking its full clinical potential. Future efforts should focus on refining viral engineering, optimizing combination strategies, and evaluating administration approaches, such as single versus repeated cycles of OAd therapy. These developments will be essential to fully harness the therapeutic power of OAds and enhance outcomes in the next generation of cancer immunotherapy.

## Author Contributions

Reza Nadafi wrote the manuscript and designed the figures. Wenliang Dong and Victor W. van Beusechem provided critical scientific advice and reviewed the manuscript.

## Conflicts of Interest

Reza Nadafi and Wenliang Dong are employees of ORCA Therapeutics. Victor van Beusechem is a named inventor on issued patents licensed to ORCA Therapeutics and owns options on shares of ORCA Therapeutics.

## Peer Review

The peer review history for this article is available at https://publons.com/publon/10.1002/eji.70024.

## Data Availability

Data sharing is not applicable to this article as no datasets were generated or analyzed during the current study.
